# The Exploration-Exploitation Dilemma: A Multidisciplinary Framework

**DOI:** 10.1371/journal.pone.0095693

**Published:** 2014-04-22

**Authors:** Oded Berger-Tal, Jonathan Nathan, Ehud Meron, David Saltz

**Affiliations:** 1 Mitrani Department of Desert Ecology, Jacob Blaustein Institutes for Desert Research, Ben-Gurion University of the Negev, Midreshet Ben-Gurion, Israel; 2 Department of Solar Energy and Environmental Physics, Jacob Blaustein Institutes for Desert Research, Ben-Gurion University of the Negev, Midreshet Ben-Gurion, Israel; 3 Physics Department, Ben-Gurion University of the Negev, Beer Sheva, Israel; Brain and Spine Institute (ICM), France

## Abstract

The trade-off between the need to obtain new knowledge and the need to use that knowledge to improve performance is one of the most basic trade-offs in nature, and optimal performance usually requires some balance between exploratory and exploitative behaviors. Researchers in many disciplines have been searching for the optimal solution to this dilemma. Here we present a novel model in which the exploration strategy itself is dynamic and varies with time in order to optimize a definite goal, such as the acquisition of energy, money, or prestige. Our model produced four very distinct phases: Knowledge establishment, Knowledge accumulation, Knowledge maintenance, and Knowledge exploitation, giving rise to a multidisciplinary framework that applies equally to humans, animals, and organizations. The framework can be used to explain a multitude of phenomena in various disciplines, such as the movement of animals in novel landscapes, the most efficient resource allocation for a start-up company, or the effects of old age on knowledge acquisition in humans.

## Introduction

In order to produce high quality science, a scientist needs to be well versed in theory and familiar with other studies in her or his field. However, spending too much time delving into other studies might reduce the time allocated to the scientist’s own research, reducing the quality of the research’s results. Assuming the scientist wants to maximize his/her contribution to science, how much time should he/she spend on acquiring knowledge vs. putting this knowledge to use?

The trade-off between the exploration of new possibilities and the exploitation of old certainties constitutes one of the most basic dilemmas that both individuals and organizations constantly face at multiple time-scales, and has therefore been investigated by researchers from a variety of fields, including economics [1]–[3], business management [4], [5], psychology [6], [7], computer sciences [8] and ecology [9], [10]. This dilemma stems from the fact that gathering information and exploiting it are in many cases two mutually exclusive activities. These two activities can be viewed as the two extreme strategies at the ends of a continuous scale. At one end of the continuum, an individual or system that only explores (i.e., obtains information about its environment in order to enhance future performance [11]) will pay the costs of obtaining new information without gaining the benefits of knowledge [2]. On the other end of the continuum, an individual or system that only exploits (i.e., uses existing knowledge only) will lack the capability to adapt to significant environmental changes and may be trapped in a suboptimal stable equilibrium [2], [4]. Thus, optimal behavior usually requires some balance between exploratory and exploitative behaviors [2], [9], [10].

Most of the studies dealing with the exploration-exploitation tradeoff show optimal solutions that are composed of one or several stationary strategies [12]. These could be a point on the exploration-exploitation continuum representing a division of the subject’s resource allocation between exploratory and exploitative behaviors that yields the best long-term rewards under given conditions [13], [14], or a point in time in which the subject should switch from a purely explorative strategy to an exploitative one [14], [15]. A more realistic approach should consider the strategy itself as a dynamic component that varies with time in order to optimize a definite goal, such as the acquisition of energy, money, or prestige. If we take the scientist from the opening example, it is reasonable to assume that his/her optimal strategy as a graduate student should differ considerably from his/her optimal strategy once he/she received tenure. Therefore, a key question is how will the optimal solution change with time along the different stages of the scientist’s career? Only very few studies have explored this optimization problem.

The principles of reinforcement learning (RF) theory, a framework originally used for machine learning that is aimed at facilitating adaptation to an environment based on trial and error [8], were applied in computational biology to construct learning algorithms in which an agent can control the balance between exploration and exploitation in an optimal manner [16]–[18]. These algorithms are based on a Bayesian modeling approach where the agent’s decisions are the product of a weighted average of some prior knowledge regarding the environment and current sampling information [19], and the agent’s need to explore is directly based on its perception of the environment, growing whenever the environment changes [16]. This is due to the fact that uncertainty should promote exploration [20] in an attempt to reduce it, and indeed there is evidence that surprising events and changes to the environment promote animals to learn faster [21]. Such algorithms have been tested and found to produce near optimal results in simulations. Moreover, analogical neurophysiologic pathways in the brain of animals and humans have been suggested, highlighting the neurobiological substrates that are related to the regulation of decision-making [17], [18], [20]. But although RF models are very useful in increasing our understandings of how animals and humans make decisions, they are also very mechanistic in nature and are, in many cases, specifically tailored to solve certain tasks, such as passing through mazes [16], with no attention given to the general motivation and ecological background of the subject. In other words, the abovementioned models have concentrated on the *how* rather than on the *why* of the decision-making process. Furthermore, so far the conclusions of all previous investigations of the exploration-exploitation dilemma are restricted to the discipline in which the study was conducted, and no attempt has been made to create a unifying framework that would be applicable across disciplines.

We present a multidisciplinary general framework of the exploration-exploitation trade-off, motivated by a new mathematical model, in which the balance between exploring new possibilities and exploiting old certainties varies dynamically with time to optimize a predefined goal. In this framework we focus on the optimal exploration-exploitation strategies at different stages of a subject’s life-span.

## Methods

Our model depicts a subject that can invest in energy acquisition (exploitation) or knowledge acquisition (exploration), according to a strategy that represents the proportion of time the subject invests in knowledge acquisition as a function of time along its lifetime *T*_max._ Denoting the subject’s energy and knowledge by *E* and *L*, respectively, and the time dependent strategy by *u(t),* the model reads:


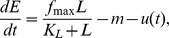
 
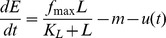


According to this model, energy *E* is gained as a saturating function of the existing knowledge *L*, with the half saturation constant *k_L_*, so that an increase in knowledge yields a smaller increase in energy gain when existing knowledge is higher. The constant *k_L_* can also represent spatial unpredictability – a low value of *k_L_* reflects a homogeneous environment in which a low amount of exploration is all the subject requires in order to gain benefits from it, while a high value of *k_L_* represent a heterogeneous environment. Energy is lost due to maintenance costs at a constant rate *m*, and also due to knowledge acquisition at a rate proportional to the strategy *u(t).* Knowledge gain is proportional to *u(t),* with efficiency *α*, and knowledge loss due to maintenance costs is proportional to the existing amount of knowledge with a rate *m_L_*. A high value of *m_L_* (i.e., a high rate of knowledge loss or “forgetting”) can represent low temporal predictability in the environment or, alternatively, the subject’s limited ability to retain stored knowledge. To obtain physically feasible results, we must also add constraints requiring that energy will not become lower than some minimal level needed for survival (*E*_min_), and also enforcing positive values of knowledge throughout the simulation:



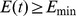


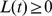



We also require the strategy *u(t)* to be limited by the following constraints: Energy expenditure for exploration, per unit time, cannot have a negative value and should be smaller than the maximal energy acquisition rate *f*_max_.



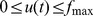



Table 1 lists the different parameters used in the model, the range of values which we investigated for each parameter, their units, their meaning, and the initial conditions and constraints of the model.

**Table 1:** The different parameters that were used in the model and the range of parameter values we investigated (A), and the parameters that were used in solving the optimization problem (B).

A. Model Parameters			
Parameter name	Values	Units	Meaning
*f*_max_	[0.5–10]	*E/t*	Maximal energy consumption rate
*k_L_*	[0.001–10]	*L*	Efficiency of foraging: The level of knowledge that will yield half of the maximal consumption rate.
*m*	0.02	*E/t*	Maintenance cost of living
α	[0.5–10]	*L/E*	Efficiency of learning: Knowledge gain per unit energy.
*m_L_*	[0.01–1]	1/*t*	Knowledge maintenance cost (temporal predictability)
*T*_max_	[5–100]	*T*	Life duration
**B. Optimization problem parameters**			
**Parameter name**	**Values**	**Units**	**Meaning**
*E*(*t = *0)	5.5	*E*	Initial energy
*L*(*t = *0)	0	*L*	Initial knowledge
*E*_min_	5	*E*	Minimal energy for survival
*L*_min_	0	*L*	Minimal knowledge
*U*_min_	0	*E/t*	Minimal investment in learning
*U*_max_	1	*E/t*	Maximal investment in learning

Each strategy, *u(t)*, correspond uniquely to a value of energy at the end of life, *E_i_(T*_max_ *)*.

We define the optimal strategy u^*^(t) to be the strategy that maximizes the amount of energy at the end of the subject’s life-span, *T*_max._ This does not mean that the subject ends its life with stores of wasted energy, since this energy is presumably used during its life-span to produce offspring, increase the subject’s material wealth, etc. In order to find such optimal strategy one can transform the optimization problem above to a set of differential equations. The rules to make this transformation were formalized by Lev Pontryagin and Richard Bellman, and are now widely known as Optimal Control Theory [22]. The differential equations obtained by this method are often quite complicated to solve analytically and may require the use of numerical solution methods. In this work we use an optimization problem solving code for MATLAB (version 7.6.0, MathWorks, Natick, Massachusetts) called “*General Pseudospectral Optimization Software (GPOPS)” available freely online* [23]. This code transforms the model, constraints, and optimization criteria using the optimal control scheme into a set of partial differential equations, and proceeds to solve these equations using a numerical pseudospectral method. The solution yields the optimal strategy u*(t) that corresponds to the maximal energy gain during lifetime. We used this method iteratively to explore how changing model parameters affect the optimal strategy.

As in all models, we make several simplifying assumptions in the construction of this model. We assume that all parameters remain constant throughout a subject’s life-span, as well as the value of information. We also assume that the rate of learning is reduced with the accumulation of knowledge. We believe that while these assumptions imply that the model may not apply to some specific cases, they also keep the model general enough to be applicative across disciplines.

## Results and Discussion

**Figure 1:**
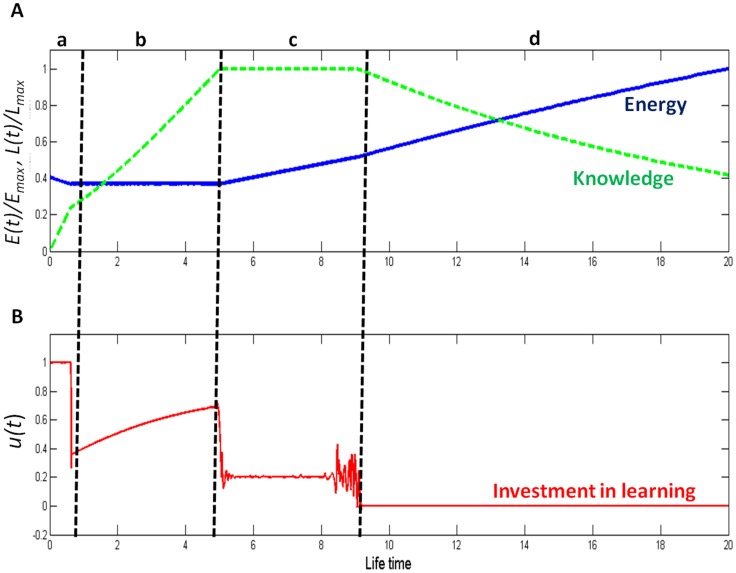
The four knowledge phases. The change with time in the subject’s energy state (*E*; panel A, solid blue line), knowledge state (*L*; panel A, dashed green line), and its optimal proportion of time devoted to knowledge acquisition (u*(t); panel B, solid red line). The vertical dashed lines make a distinction between the four life-phases with regards to the exploration-exploitation dilemma: *a*. Knowledge establishment. *b*. Knowledge accumulation. *c*. Knowledge maintenance. *d*. Knowledge exploitation. The parameters used to generate this example are: *f_max_*_ = _1, *k_L_* = 1, *m_L_* = 0.08, *alpha* = 1 and *T_max_* = 20.

The model results were very robust, and remarkably produced only four distinct phases that emerged in a fixed order regardless of the parameter values that were assigned. The phases differed in the subject’s relation to knowledge (Fig. 1) and can be defined as: 1. Knowledge establishment. 2. Knowledge accumulation. 3. Knowledge maintenance. 4. Knowledge exploitation. Each of these phases relates to a different stage in the life-span of the decision making subject, be it a foraging animal, a human or a company. The framework is relevant across disciplines and can be used to explain a multitude of phenomena and allow for better informed decision making.

## The Four Knowledge Phases

### Knowledge Establishment

In order to exploit any resource, even in the most inefficient manner, the exploiting entity must have some knowledge of its environment. At the very least, knowledge of the existence of a resource and how to reach it are needed. The more is known about alternative resources, ways of obtaining them and various aspects of the environment, the more efficient the exploitation of resources will be. Thus, *knowledge establishment* is an obligatory phase when entering unfamiliar territory, such as for a dispersing or translocated animal, or an emerging company.

During this phase the subject devotes all of its resources to exploration (Fig. 1). Since the subject does not exploit any resources, it relies solely on its internal reserves (i.e., the energy state of an exploring animal or investors’ funds in an emerging company). Consequently, the length of this phase is mainly determined by the subject’s initial state. A subject that is in a relatively good state can afford to extend this phase considerably, thus improving its future prospects.

It is important to note that both humans and animals frequently use inherited knowledge (that was passed to them genetically or through culture transmission) when entering an unfamiliar territory, and thus may act upon some prior expectations based on that knowledge. If this knowledge is reliable, these individuals may skip this phase entirely and start their life from the knowledge accumulation phase. However, inherited knowledge may sometimes hinder the utilization of resources [24], such as in the case of rapidly changing environments, in which case individuals may be left with diminished resources for the establishment phase.

This phase is commonly apparent in technological ventures where in the early stages of a development project, an exploratory search should be undertaken in an attempt to discover something new, as well as to form exploration alliances [5], [25]. In the context of animals, this phase exists in dispersing individuals that have reached unfamiliar territories. It is usually very short, and thus there is very little empirical work investigating it in the wild. However, we do know that captive animals that are introduced to new environments exhibit specific behaviors aimed at exploring their new environment [26], [27]. The rapid integration of high resolution GPS collars into wildlife reintroductions [28] promises exciting advances in this field, as we now have the means to investigate the movement behavior of animals that are released to novel environments to better understand the knowledge establishment phase.

### Knowledge Accumulation

This phase is what most literature dealing with the exploration-exploitation trade-off refers to as the exploration stage. During this phase the subject focuses on obtaining new information while exploiting resources from existing knowledge at a low rate aimed only at keeping the subject at some minimal pre-defined state. Thus, the subject is sacrificing its short-term benefits in order to obtain long-term rewards. As this phase progresses the rate of obtaining new information increases slowly because with the accumulation of knowledge, the exploitation of existing resources becomes more efficient and the subject needs to devote less time and energy to reach its minimum pre-defined state, and can therefore allocate more time and energy for further exploration (Fig. 1).

Since exploratory behavior is such a fundamental behavior in both humans and animals [29], there have been many attempts to describe and characterize the behavior of individuals in novel environments. Some of the more in-depth studies of exploratory behavior have been done on rodents, but even within these studies, exploratory behavior varies according to the species and context. Laboratory mice introduced to a novel arena, showed exploratory behavior of increasing complexity, first examining their nest’s surroundings, then progressively the walls around the arena and only later venturing to the center of the arena [29]. A similar behavior was performed by fat sand rats, *Psammomys obesus*, under lit conditions, but in the dark the rats performed looping behavior, in which travel paths tangle into loops [26]. Outside the laboratory, brown rats, *Rattus norvegicus*, released into the wild, exhibited random walk patterns, increasing in perimeter with time and mediated by central place foraging behavior [30]. Whatever the exploration method is, in all of these cases the behavior of the animals is clearly primarily aimed at increasing their knowledge about their surroundings and not at the acquisition of resources. Thus, all of these different exploration mechanisms ultimately represent the same phase – *knowledge accumulation*.

**Figure 2:**
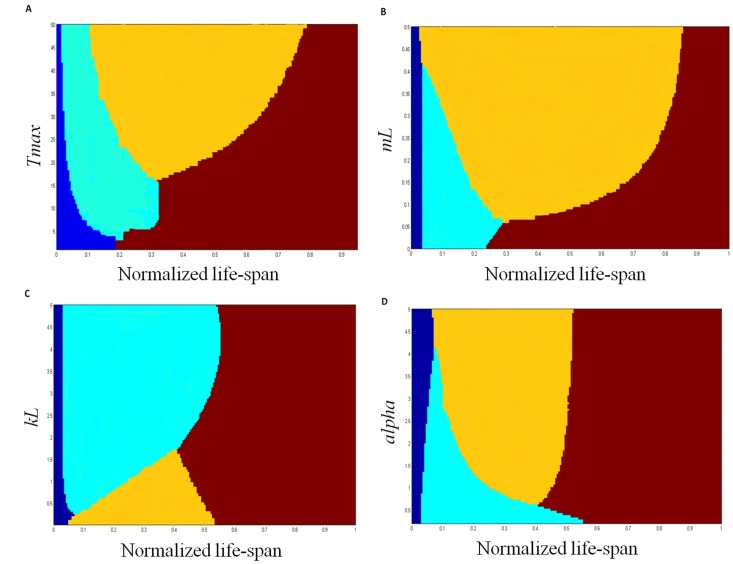
The optimal knowledge phases as a function of age and environment. The four optimal knowledge phases (dark blue - knowledge establishment, light blue - knowledge accumulation, orange - knowledge maintenance, red - knowledge exploitation) as a function of the subject ‘age’ (i.e., its position on its life-span trajectory, normalized here to a scale of 0–1), and different parameter values: (A) *T_max_* - length of life-span. (B) *m_L_* - rate of knowledge loss. (C) *k_L_* - learning half saturation constant representing the environmental spatial predictability. (D) *alpha* - learning efficiency. In all simulations, the values of all parameters not tested (e.g., for plate A - all parameters but *T_max_*) are as described for figure 1.

The subject’s time horizon (*T*_max_) is an important factor determining the length of this phase. Because there is a temporal gap between paying the short-term costs of accumulating knowledge (i.e., exploring) and reaping the benefits of information, subjects with short life-spans should invest less in accumulating knowledge, since for them the benefits of knowing more are greatly reduced. Indeed, numerous studies on humans and animals report that as the relevant time horizon decreases, so does the tendency of the subject to explore [9], [18], [31]. A limited time horizon can stem from the time left available for a specific task [32] or the age of the subject [33]. Increasing the time-span of a learning subject will lengthen the *knowledge accumulation* period, but only up to a certain value. Because of cognitive or physiological constraints, as well as environmental stochasticity (that in most cases cannot be fully predicted), there is a limit to the benefits of exploration. Thus, eventually the exploring subject reaches a point in which additional exploration does not improve its future prospects and this phase becomes constant (decreasing the relative weight of this phase as the subject’s life-span increases, Fig. 2a).

The environment’s temporal unpredictability (*m_L_)*, which can reflect either external conditions that change with time (such as a highly fluid market environment), or the subject’s own cognitive abilities and liabilities (such as memory capacity or decay), will also determine the length of the knowledge accumulation period. The more unpredictable the environment is, the harder it is to make predictions about the future state of the environment, which lowers the value of exploration (Fig. 2b). This result is supported by both theoretical models of learning in stochastic environments and empirical studies with humans [20], [34], [35].

As the spatial unpredictability (*k_L_*) of the environment decreases (i.e., as the environment becomes more homogeneous) the need for exploration is reduced, and in extremely predictable conditions the knowledge gained during the *knowledge establishment* period is sufficient for optimal exploitation, eliminating the *knowledge accumulation* phase (Fig. 2c). Lastly, the learning efficiency (*α)* of the subject will determine the length of the *knowledge accumulation* period. An extremely efficient learner already accumulates enough knowledge during the knowledge establishment period, and can skip the accumulation stage altogether. In contrast, for an inefficient learner the accumulation period is greatly extended to allow for the accumulation of sufficient information for optimal exploitation of resources at a later stage (Fig. 2d).

### Knowledge Maintenance

In this phase the subject focuses on the utilization of resources while maintaining its knowledge at a constant optimal level. i.e., learning is only used to replace lost information or update existing knowledge. The leveling of the knowledge curve (Fig. 1) represents an optimal level of knowledge. Obtaining additional knowledge is too costly (because of the saturating shape of the energy gain function) when weighted against the benefits of knowledge and the rate of knowledge loss (*m_L_*).

For animals foraging in heterogeneous landscapes with renewable resources, trap-lining, defined as repeated visitation to a series of resource patches in a predictable order, is usually the most beneficial foraging strategy [36], and has been reported for a wide variety of species [37]–[39]. Trap-lining foragers utilize resources based on existing knowledge, but since the environment is constantly changing, some method of updating the forager’s information regarding its environment is needed for it to avoid getting ‘stuck’ in an inefficient foraging route. Indeed, several cognitive mechanisms for updating trap-lines have been suggested [10], [36]. One suggested mechanism that can control both this phase as well as the *knowledge accumulation* phase is the adding of a (usually positive) bias to the subject’s estimation of its environment when it encounters a novel environment (or alternatively, the adding of stochastic variability to its estimate). This idea originates from the field of RL and machine learning [8], [40], but has lately been expanded to explain animal behavior [10], [41]. A positively biased estimation of the environment encourages exploration by motivating the subject to keep looking for better rewards. As the subject explores, it constantly updates it estimate of the environment reducing its initial bias. Thus, the longer it explores, the more realistic this estimation will become, until eventually the subject will cease exploration and move into the knowledge maintenance phase. The same mechanism will also ensure that the subject maintains its knowledge in the maintenance phase. Either that stochastic error in the subject’s learning mechanism will keep him exploring to some degree throughout this phase, or alternatively, in the case of an initially biased estimation, whenever the subject encounters a lower than usual reward, as a result of some degradation in the quality of the familiar environment, it will again possess an estimate that is higher than the rewards it acquires, which will send him exploring for a better alternative.

In business management, during the *knowledge maintenance* phase, knowledge regarding existing products is used and maintained, but new lines of products are not pursued [2], [14]. The maintenance of knowledge is essential to effectively manage the inevitable errors and changes that are associated with knowledge storage bases, and is therefore considered an essential element of knowledge management [42].

Just as in the *knowledge accumulation* phase, a short time horizon will reduce the length of the *knowledge maintenance* phase, or even eliminate it altogether (Fig. 2a). When the subject’s time-span is very short, it will be sub-optimal to spend any time learning new information, even if only to maintain the subject’s current knowledge. However, unlike the *knowledge accumulation* phase, as the time-span of the subject expands so does the amount of time devoted to *knowledge maintenance*. During this phase the subject reaps the rewards of past explorations, and thus the longer this period lasts, the more the subject gains.

This phase is strongly affected by the environment’s temporal unpredictability. In an environment that is predictable (as a result of stable conditions and low memory decay of the subject) this phase diminishes as the knowledge that was acquired earlier does not need maintaining and the subject should focus only on exploiting it. On the other hand, in a very fluid (and hence, unpredictable) environment, this phase replaces the *knowledge accumulation* phase simply because there is no point in accumulating knowledge for future use in a constantly changing environment and the subject should focus on continuous learning while exploiting resources (Fig. 2b). The learning efficiency of the subject produces a similar trend - when it is very low, there is no use in trying to maintain knowledge, since the benefits of investing only partial efforts in learning are close to nil. In this case the subject should concentrate only on the exploitation of knowledge once its *knowledge accumulation* phase is over. When the learning efficiency is especially high the amount of resources devoted to learning during this phase can be maintained at a very low level, and it can replace much of the *knowledge accumulation* phase (Fig. 2d).

### Knowledge Exploitation

This phase arrives towards the end of a subject’s life-span, and is characterized by a learning investment of 0. As the end approaches, it is sub-optimal to continue investing in gaining new information and the subject should invest its time only in exploiting the knowledge it had already accumulated, temporarily increasing its intake rate of resources (Fig. 1). It is worthwhile to note that in most cases a subject will have no prior information on its expected life-span. However, there are usually detectable cues that can inform the subject it is approaching the end of its life.

We do not presume to suggest a mechanistic explanation to the effects of old age on learning performance. However, from an evolutionary perspective, our framework corresponds to several of the main paradigms of the psychology of human aging. It is common knowledge that the processing of information and memory in humans decay in old age [43]. Moreover, in respect to reading, older subjects show a substantial decline in their working memory, but an increase in their use of prior knowledge [44]. Three processing styles have been identified in relation to age [45]: The ‘youthful’ style focuses on learning, intense data gathering and bottom-up processing. The ‘mature’ style balances the use of relevant knowledge and information seeking, and the ‘old’ style relies on top-down processing, making use of existing knowledge. This notion that aging is accompanied by an increase in top-down processes pervades recent literature on language in old age [46], [47].

Another popular theory that supports our framework is the Socioemotional Selectivity Theory [31], [48], [49]. The theory proposes two primary motivations for social interactions: emotion regulation and knowledge acquisition. The perceived time-span of an individual determines the relative importance of these motivational objectives. A long time-horizon tends to be related to knowledge acquisition goals, while a limited time-horizon tends to be related to emotion regulation goals. Because of their limited future time extension, older adults are assumed to be less motivated to acquire knowledge. The theory has received empirical support in a variety of studies [50], [51]. While this can also be explained by the biological fact that the cognitive abilities in humans decay in older people, empirical evidence demonstrates that young people with a limited time horizon (such as terminally ill patients) show similar tendencies to forgo knowledge acquisition [51], [52].

It is interesting to note that for very short *T*_max_ only two phases emerge - knowledge acquisition and knowledge exploitation. Animals with very short life-spans are usually also very small (as they do not have the time to invest in a large body). Small size and a short life-span may promote a more homogeneous environment in space and time (e.g., the animal only lives through one season and forages in a single habitat), which means that there is no need to maintain the knowledge and once enough knowledge is acquired, the animal can immediately switch to the exploitation of resources with no further investment in learning. As lifetime increases, animals need to deal with a more complex environment (more seasons, more habitats), and thus knowledge accumulation and maintenance stages are added to their life-time strategy.

## Conclusions

We provide a unifying framework of the exploration-exploitation trade-off, a trade-off prevalent in many disciplines and situations. It is important to note that the timeline presented in our model is restricted to monotonic linear time changes (e.g. lifetime of a human; lifetime of an economical project). However, the model could be easily extended to account for non-linear time-frames. For example, a major change to the environment (e.g., a flood that changes the entire topography, or an economical crisis that changes the entire economical landscape) can force a subject to revert from the *knowledge maintenance* or even the *knowledge exploitation* phases back to the *knowledge accumulation* or *knowledge establishment* phases. Similarly, there can be cases in which the entire sequence of 4 phases can occur multiple times within a subject’s life-span, such as in the case of animals that disperse to new areas several times during their lifetime. In such cases, the length of each sequence can change with time and ‘dispersal experience’, i.e., the explorative phases of an animal dispersing for the first time may be considerably longer than for an animal dispersing to an unfamiliar area for the fifth time in its life.

Our framework demonstrates that the optimal solution to the exploration - exploitation trade-off depends on the life-stage of the subject as well as on the environmental conditions, and that the same strategies can be used by a variety of subjects - animals, humans and organizations alike. This fact points to the universality of the exploration-exploitation dilemma and the strategies aimed at solving it. Thus, the proposed framework can improve our understanding and consequently, our decision making in a multitude of disciplines.
