# Correction: The Exploration-Exploitation Dilemma: A Multidisciplinary Framework

**DOI:** 10.1371/journal.pone.0119116

**Published:** 2015-03-05

**Authors:** The PLOS ONE Staff

In the Methods section, there is an error in the first equation in the first paragraph. Please view the complete, correct equation here:

$$\frac{dE}{dt}=\frac{f_{\max}L}{K_{L}+L}-m-u(t),\frac{dL}{dt}=\alpha u(t)-m_{L}L$$
